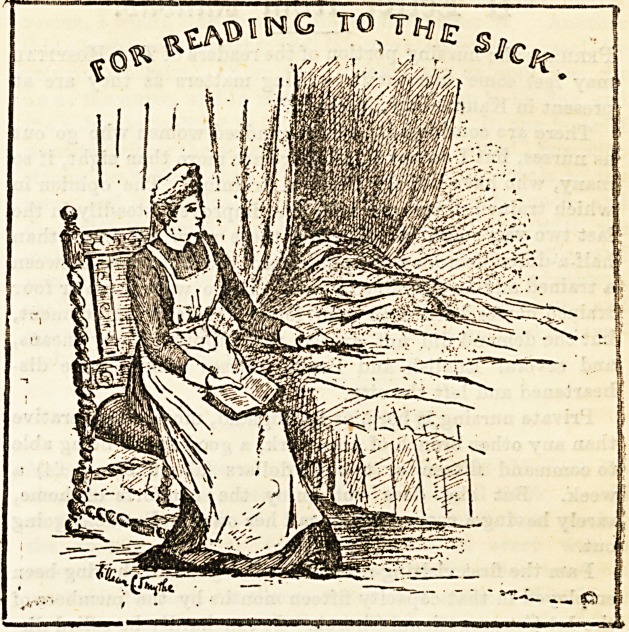# The Hospital Nursing Supplement

**Published:** 1891-05-09

**Authors:** 


### "The Hospital" Nursing Mirror.

Being the Extra Nursing Supplement of "The Hospital" Newspaper.

Contributions for this Supplement should be addressed to the Editor, The Hospital, 140, Strand, London, W.C., and should have the word "Nursing" plainly written in left-hand top corner of the envelope.

### En Passant.

South London Association.—The district nurses of South London are under very distinguished patronage just now. On Saturday last a concert was given on their behalf at Grosvenor House when Lady Ramsay of Banff, Lord Compton, and others sang. On June 25th, 26th, and 27th a grand midsummer fete in aid of the same association will be held at Bramblebury, Wandsworth Common, when there will be Pastoral Plays, out-door sports, and all kinds of charming amusements. There will be prize competitions and exhibitions of dressed dolls, millinery, carving, floral decorations and decorative art.

Short Items.—Miss Kate Marsden has arrived at Irkutsk, Siberia, on her way to Yakutsk, for the purpose of studying leprosy among the natives.—Mrs. Stuart-Wortley is helping to start a district nurse at Kirkheaton.—Jedborough is to have a "Queen's nurse."—A sale of work in aid of the work of the Sisterhood of St. John the Divine will be held at St. John's Hospital, Morden Hill, Lewisham, on June 10th and 11th; the nurses will hold a stall furnished by themselves.—There will be a bazaar at Caldicote House, Bushey Heath, on May 28th, which Lady Halsbury will open.—Miss Ethel Lamport has been appointed lecturer on nursing at the Forsyth College.—Mrs. Perry has taken Redlands, Glen Fern Gardens, Bournemouth, and is prepared to receive and attend to invalids.—A Sister of Charity has been indicted at Lyons for illegally practising pharmacy; she had a favourite prescription for anæmia, and used to sell it to her friends.

Hedda Gabler.—In spite of the outpourings of opprobrium from the critics, Ibsen's last play, after two series of trial matinées has been put in the evening bill at the Vaudeville. It is worth while to mention the play here, for to a woman, more especially to a nurse, the character of Hedda Gabler, which has baffled and fooled the men is so perfectly natural and comprehensible. Hedda is a creature of fine physique; she is full of energy, her life consists of trifles. At length she marries a professor, because, as she herself expresses it, she is tired of dancing. When the play opens Hedda has been married seven months, and has just returned to her home; she is in delicate health, but every reference to her condition only irritates her. Now, all nurses and medical men know that at this time a woman should be humoured, and that she is to a large extent irresponsible for her actions. Also revulsion and fear for her coming fate sometimes make this period peculiarly bitter. Hedda behaves more or less madly; she is impatient of the pettiness which surrounds her, and longs to try issues with heaven and hell. Our contention is that this is only natural when a woman of energetic mind and body is given no outlet for her forces, save that of marriage, which soon becomes distasteful to her. Had Hedda entered some profession, become a hospital nurse for instance, she might have been a good and a great woman. Ibsen is a physiologist; he knows his subject, though we admit it is not a fit subject for the stage; still it is strange that the whole army of critics have failed to discern the clue to Hedda's eccentricities, and therefore failed to foresee the success of the play.

Royal Berkshire Hospital.—This institution issues as usual a very careful and well-considered report, and there is special mention of the progress of the nursing department. Of every branch of the nursing system of the hospital the Board has to speak with satisfaction. "Of the kind and good work done by the regular day and night nurses in the wards, by the probationers, by the lady pupils, by the private and district nurses, much indeed might be said. Wokingham, York Town, and Marlow still employ district nurses from the hospital, and from each of these centres gratifying accounts have been received of the services there rendered. At present there are twenty-eight private nurses, and with scarcely an exception their faithful discharge of duties has met with grateful recognition. Perhaps the truest testimony to their efficiency is to be traced in the urgent need that is experienced of a considerable increase to their number. The demands for the services of private nurses from the hospital have so far exceeded all power of granting them, that during the past year as many as four applications have had to be refused in the course of a single day, through the fact of every nurse being engaged. This contemplated addition to the number of nurses will necessarily involve a corresponding increase in the accommodation afforded by the hospital, whether in the form of erecting new buildings or of hiring or purchasing some suitable residence." The "Nurses' Pension Fund" has been duly appreciated, and the scheme, now in full working order, promises well; it ought to affiliate with the R.N.P.F.

Leicester Institution.—On April 26th was held the annual meeting of the Leicester Institution of Trained Nurses; the Mayor presiding. The twenty-fourth annual report stated that the Committee were glad to be again able to render to their subscribers, donors, and friends a favourable account of the work done during the past year. They had, however, to deplore the great loss the institution had sustained by the death in May last of their late Chairman, Sir A. G. Hazlerigg, Bart., who from its formation took the greatest interest in its welfare, and whose decease had been so deeply and widely felt. The district nursing, which had been very heavy throughout the year, had been carried on most satisfactorily; and the seven district nurses had been constantly employed. One thousand and two cases had been nursed gratuitously. The earnings of the private nurses had not been quite equal to those of the previous year, owing to the diminution of the staff having been unusually great from various reasons. The Committee regretted that on this account many applications for nurses had had to be reluctantly refused. Every exertion was used to secure additional fully-trained nurses; but good ones were not easily obtainable, and it was therefore necessary to still keep up the number of probationers in training, although the cost of doing so added considerably to the expenses of the institution. Two hundred and twenty-four cases of private and 19 of monthly nursing have been attended, and the nurses had invariably manifested a warm interest in their work. The staff at the close of the year was as follows: Twenty-two fully-trained nurses, five of whom were certificated monthly nurses; seven fully-trained district nurses, and eight probationers being trained, total 37, or a reduction of five as compared with the previous year. The balance-sheet showed that whereas the year began with a deficiency of £217 14s. 1d., the amount due to the treasurer at the close of the year (owing mainly to the district nursing account showing a deficiency of £76 14s. 4d.) was £177 4s.

### Lectures on Surgical Ward Work and Nursing.

By Alexander Miles, M.B. (Edin.), C.M., F.R.C.S.E.

Lecture XXIII.—PLASTER OF PARIS CASE.

Uses.—In chronic diseases of joints—for example, strumous disease, or chronic synovitis, rest to the diseased part is often obtained by enclosing the limb in a case of plaster of Paris. Not infrequently it is put on over a Scott's dressing. In the treatment of fractures of the bones of the leg also a plaster case is often found useful, as it enables the patient to be up and about, with the aid of crutches, much earlier than would be possible with any less rigid form of apparatus. In fractures, however, it should not be applied till all the swelling which is usually present has subsided, as otherwise the case becomes too slack when the limb has resumed its normal size, and no longer controls the movements of the fractured ends of bone, the result being an un-united fracture. After excision of joints, *e.g.*, knee, the limb is often put up in a plaster case, so applied as to leave a "window" through which the wound is dressed, yet sufficiently rigid to prevent any movement between the opposed ends of the bone which are wanted to unite. In disease of the hip, the case may be applied to the whole length of the leg, and then be continued round the pelvis, thus securing immobility of that joint.

Materials Required.—(1) Muslin bandages ; (2) plaster of Paris (best quality) ; (3) dextrine ; (4) boracic lint bandages ; (5) scissors ; (6) pail of tepid water ; (7) one or two old blankets or sheets.

Method of Preparing Materials.—*Bandages.*—These are made of coarse muslin, and should not exceed three yards in length. An ordinary bandage should be six yards long, but if you make your plaster bandages so long you will find that the water will not percolate all through them, and much of the bandage will be wasted. They should be from 3½ to 4 inches wide according to the size of the limb to which they are to be applied. They should be kept rolled up just like an ordinary bandage before being charged with the plaster, but when charged they must be rolled very loosely. The plaster of Paris must be of the very best quality, otherwise it will not set well, and the rigidity of the splint will be diminished. The plaster should be well dried in the oven for a few days before it is used. This is important, hence in hospital, or where much plaster is used, a supply should be constantly kept preparing in the oven, so that there may be no delay when it is needed. The case is rendered more rigid and fewer bandages are required if one part of dextrine be added to every two parts of plaster. These are thoroughly mixed and prepared otherwise as plain plaster. To charge the bandages, spread a layer of brown paper on a large flat table, and unroll part of a bandage on it. Take from a basin a handful of the prepared plaster, and with the palm of the hand rub it thoroughly and equally into the muslin, so that all the meshes get filled up with it. Roll up *very loosely* the part charged, and proceed to the next part, and so on, till the whole bandage is impregnated with plaster. The reason for rolling up the bandage loosely is that the water may easily and rapidly percolate to the very centre, and to every part of it. When tight, only the outer layers are reached by the water, and the remainder is wasted. To apply the bandages, spread one of your old sheets on the floor, and another on the bed or couch on which the patient lies. Wrap a third round yourself to avoid soiling your dress with the drippings from the wet bandages. Wash the limb with soap, water, and turpentine. Apply a boracic lint bandage neatly and evenly, avoiding crossings and creases as much as possible, from the toes up beyond the part to be encased. Carefully pad any prominences, *e.g.*, malleoli, condyles, &c., with nests of corrosive wool or boracic lint. Now count out the number of bandages you expect to require and lay them apart. You are apt to lose count if you simply pick them out of the stock box as you proceed. Eight or nine plies, *i.e.*, about a dozen bandages, are required for an adult. Five or six plies, or about nine bandages, for a child. Next put one bandage into the pail of tepid water which you have provided. How do you know when it has been long enough in the water to be thoroughly saturated? When bubbles of air cease rising to the surface the bandage is saturated. Then take it out, and after squeezing from it most of the water begin to apply it to the limb. But before doing so put another bandage into the pail so that it may be getting saturated while you are applying the first. The bandage is put on in the usual way according to the part you are covering in. When you have completed the application let the limb lie exposed for about half an hour or an hour to enable the plaster to set. If you cover up the limb before the plaster has set the blankets prevent the evaporation of the moisture and the result is unsatisfactory. You should not set the patient down beside a fire to dry the case, as this only bakes the outer layers and prevents the moisture escaping from the deeper ones. Thus the bandage remains soft, and when weight is put upon it it bends.

After Treatment.—Keep a look-out on the toes to guard against vascular interference. The patient often complains that the upper margin of the case is uncomfortable. If so, you may snip it all round with scissors and turn down the edges a little.

### Examination Questions.

Answers to the last question numbered 85, and they were all more or less correct. On the whole the best answer was by Sister Aldridge, Harlaxton Manor, to whom we have sent Ostron's "Massage and Swedish Movements." Very good answers were sent by Nurse Miriam, Nurse Florence Lang, and Nurse Fletcher, while answers worthy of mention were sent by H. K. Geary, M. A. Metcalfe, H. Cary, C. Greig, J. Torrance, Nurse Paton, A. Yarrow, Mary Brown, M. L. Warneford, D. Mallins, C. Howell, F. U. L. Jenkins, J. Boyd, E. M. Williams, A. Giffen, C. Rea, Evelyn de Vos, E. Braybrooke, A. Bridger, Nurse Porter, M. C. Telford, and B. Wherrit.

The question for this month is, "What are the special dangers in convalescence from typhoid and scarlatina?" Answers must be short, and written on one side of the paper only ; they must reach this office (140, Strand) by May 23rd, and each answer must be accompanied by the writer's full name and address. Below is the prize answer for last month :—

Disinfecting after Scarlet Fever.—Bedding, and all large articles that cannot be thoroughly done at home, should be sent to the "disinfecting oven" (such places are provided by the health authorities, and they fetch the articles when applied to). There they are baked at a temperature of not less than 220 deg. F. for some hours. Clothing and all materials in the room should be hung up so as to be well exposed. All drawers, closets, and cupboards should be left wide open for the gas to penetrate to every corner. Any metal work should be coated over with grease, as the gas discolours it. Doors, windows, and fireplaces should be well closed, and all the cracks around them pasted over with brown paper. Either sulphur or chlorine may be used for fumigation. Sulphur is most common, perhaps. Take half a-pound or more of sulphur, according to the size of the room, the proportions being two pounds to every 1,000 cubic feet, break it into small pieces and place it in an iron pan, rest the pan firmly on a pair of tongs over a pail of water, as a precaution against fire ; set light to it and leave the room immediately, closing the door and pasting over the crevices outside ; leave the room closed for twenty-four hours, after which time the doors and windows may be opened wide for another twenty-four hours. Then all should be cleared up, washing materials should be boiled, and everything—the floor, woodwork, and walls if possible—should be scrubbed with bichloride of mercury (corrosive sublimate). The room must then be repapered and the ceiling whitewashed.

### Nursing Medals and Certificates.

THE KHEDIVE'S BRONZE STAR.

THOSE nursing sisters who worked through the Egyptian Campaign in connection with the English military service received from the Khedive his order of the Bronze Star, pictured above. The order has also been given to some French nursing sisters. Amongst the English holders of the Star for 1882 are Sister H. King, Sister J. A. Gray, and Sister J. M Wallace. Amongst holders of the Star for 1884 6 are Sisters A. C. Yardley, M. C. Jerrard, S. F. Hart, M. Barker, L Byham, J. King, H. C. Norman, R. Williams, A. Hind, L. Parsons, M. C. F. K. Cole, S. J. Brown, C. F. Irving, R. M. Burleigh, E. Wright and Superintendent S. Ireland. Several of these ladies have married and changed their names, for instance Miss Williams is now known as Mrs. Norris, and Miss Irving has married a doctor and is living in India.

[The previous medals and certificates given in this series are the Royal Red Cross, December 6th, 1890; Charing Cross, December 20th, 1890; Mary Adelaide Medal, January 3rd, 1891: Butterworth Medal, January 24th; Kent Nursing Institution, January 31st; Liverpool Nurses' Training School, February 7th: Suakim Medal, February 21st: The London Hospital, March 7th; St. Bartholomew's Medal, May 2nd.]

### Notes and Queries.

### Queries.

(8) *Smell of Sulphur.*—How can I get rid of the smell of sulphur from a feather bed and pillows, fumigated for disinfection?—H.

### Answers.

*Nurse Annie.*—We cannot advise you where to apply for nursing work, save to answer the advertisements in our pages. As for going abroad, it entirely depends on your introductions, training, and perseverance. Some nurses do well—see letters from America and Australia we have published—others do ill.

*A. A., Harrogate.*—Thanks for your suggestion, but we fear our readers would find it monotonous. The photographs of the screen are so clear, that most of the likenesses can be recognised.

*Charge Day Nurse.*—Certainly the charge nurses are all equal, whether on day or night duty. Indeed, the night nurse generally gets higher pay. You have been misinformed.

(5) *An Epileptic Case.*—Apply to the County Asylum; they all have wards for idiots and epileptics.

(6) *Nursing Ethics.*—It is usual for a Matron to inform her Committee when she is seeking another post; indeed, it is necessary to do so to secure their testimonials.

*Hannah.*—Cullingworth's "Manual for Monthly Nurses," published by Churchill, price 1s. 6d.

*R. M.*—Apply to Messrs. Shoolbred, of Tottenham Court Road; they supply cloaks, bonnets, and veils. Or you might apply to Whiteley.

*Lady Superintendent.*—We have noted the cases sent, with thanks; we shall proceed with the matter shortly.

*Nurse C.*—The list is posted at the office of the L.O.S., Berners Street, and the Midwives' Institute, Buckingham Street, Strand; it is printed in *Nursing Notes*

*E. M. S.*—Testimonials are usually returned as soon as an engagement is made; no nurse ought ever to part with her original testimonials.

FORGIVENESS.

WHAT a generous standard the Gospel gives us about forgiving our enemies. We are to do so, not seven times only, but seventy times seven, which really means the forgiveness of every offence that is committed against us without limit to the number of times, or the seriousness of the faults.

At present you have time to think over such things and recall whether you yourself might not have made the first step towards some quarrel which has parted you from your friend, and if not, whether you gave to the sharp, hasty word the soft answer which turneth away wrath. What a happy life ours would be if there were no tiffs or misunderstandings, but we are all so fond of our own way that we cannot bear the least opposition. Even when we are quite in the right we often nurse unkind thoughts about the offender, whereas if we went and told him gently of his fault *alone*, quite by ourselves, we should most probably gain our brother's love back. Our duty is put so plainly and easily before us in the Sermon on the Mount that it is strange we do not carry it out. We ought to be afraid to use the Lord's prayer and ask God to forgive us our trespasses if we do not forgive those who trespass against us. We must forgive for Christ's sake every injury or wrong done to us and not stand upon our rights, but accept the very smallest offers of reconciliation. We must forgive and forget too.

You say it is very hard to do either. It may be so and will be, till we can realise the tender love of Him who died that our sins and offences might be blotted out. The more we love Him the more we shall see how hard and unkind and vile is our natural heart, and what a fearful debt we owe our Creator. But being freely forgiven we will learn how freely to forgive.

### Death in Our Ranks.

WE announce, with regret, the death of Nurse Wilhelmina M'Arthur, of the Netherfield Road Hospital, Liverpool. Nurse M'Arthur had been on duty in the typhus wards for six months, and contracted the disease while performing her duties.

FROM abroad we learn the death of two distinguished nurses, Froken Louise Couring, of the Copenhagen Deaconess Home, who founded trained nursing in Denmark; and Fru Velly Velander, wife of the celebrated oculist, and nurse to many of his poor patients.

### A Letter from Kansas.

Perhaps the nursing portion of the readers of The Hospital may feel some interest in nursing matters as they are at present in Kansas City, Missouri.

There are considerably over a hundred women who go out as nurses, but I do not think there are more than eight, if so many, who have had any hospital training. The opinion in which trained nurses are held has improved steadily in the last two years and a-half, when there were not more than half-a-dozen doctors who appreciated the difference between a trained and an untrained nurse. There were three or four trained nurses here then who found constant employment, but the demand did not exceed the supply by any means, and several English and Boston nurses soon became disheartened and left the city.

Private nursing is here, as in England, more remunerative than any other branch of the work, a good nurse being able to command fifteen or twenty dollars (about £3 or £4) a week. But she does not enjoy the comforts of home, rarely having a room she can call her own and seldom going out.

I am the first visiting-nurse K— C— has had, having been employed in that capacity fifteen months by the members of the 1st Congregational Church. It can hardly be called district nursing as I go all over, and sometimes quite a distance outside the city. We have a well-stocked loan-closet, and also a delicacy-closet, containing, as its name implies, delicacies, supplied by the ladies of the Church, and which I carry to the patients. The first few months were not encouraging; it was so difficult to make the physicians understand the kind of work we proposed doing, or to get them to send us cases, though we supplied them with directed post cards. The statistical report for the fifteen months is as follows:—Number of cases, 143; number of visits, 2,525; number of articles lent, 410; number of articles lost, 16. The number of visits includes many of investigation and where other than nursing help has been extended, my work not having been so far strictly confined to nursing. I have begged at different times for everything that mortal is ever in need of, from the first suit of baby-clothes (we have a stock always on hand now) to money to defray funeral expenses. The charities here are not thoroughly organized and leave much to be done by individuals.

I have had a number of coloured patients of all ages, and, contrary to everything I had previously heard, found them in many instances, given the same conditions, superior to white people in cleanliness and general thrift. Their inherent love of finery seems to fire them with an ambition seldom seen in the lower classes of our own race.

Some idea of the state of the roads may be formed from the fact that those who have to brave all kinds of weather wear rubber boots reaching to the knee. A few days ago I went down nearly to the top of mine.

Since I came we have not had a severe winter, but I have had to stand before the fire to thaw the frozen rain off my coat before I could remove it. And imagine pursuing one's daily work when the thermometer registers anything from 104 deg. to 124 deg. But the springs and autumns are glorious, and in spite of sudden climatic changes Kansas City is a wonderfully healthy place, the late death returns reporting only 15 per 1,000.

The hospitals, of which there are six, including two rail-road —used exclusively for accidents occuring on those particular lines—two Catholic, one High Church, one general, and one children's, are very poor, excepting the latter, which, though small, is conducted on the principles of the Eastern and English hospitals. The general, or as it is called, City Hospital, is a wretched place, but, alas! the doctors seem satisfied with it, and there are only a few enterprising souls who take the trouble to compare it with other institutions of the kind, and long for the time when ours shall vie with the best.

Q.

### Private Nursing.

"It does not matter where she is sent, she is always the right person in the right place," said a Lady Superintendent, speaking of one of her private staff, and no better words could be found to describe that valuable member of the community, a really good private nurse. Adaptability is one of the most important, if not the greatest of her virtues; she must be able to seize the situation at a glance, and, putting aside mere personal desires and antipathies, must set herself with a cheerful face to smooth down the rough places, and to ignore the little pricks that are so hard to bear, and that will turn up even at the easiest and pleasantest cases.

In hospital work there are certain fixed rules which patient and nurse are bound to observe; in private work the nurse must rely on her own judgment, and on personal tact to carry out many things on which the success of the treatment and the patient's comfort depends. The doctor relies on his trained help to keep the invalid from worrying over trifles, to see that surroundings are in accordance with the well-being of the patient, and that friends do not weary him by too frequent visits. In many cases the doctor will leave no strict rules as to diet; he will depend on the nurse to find out what tempts the appetite and suits the patient's digestive powers. In chronic and minor cases the visits of the medical man occur irregularly, and often at long intervals, and it is imperative that the nurse should be able to use her own judgment, and to act with promptitude in the small emergencies that crop up daily. Among her greatest trials are the friendly visits which, if paid by the right people, help to brighten and shorten the invalid's day. But it often happens that after the visitor's departure the patient is found with a bounding pulse and excited brain, or depressed and faint because he has been told some item of family news that should have been delayed till stronger days. We learn to dread as much the friend who comes with a laughing face to keep up the invalid's spirits, and who excites the poor weak nerves with comic anecdotes, as we do the mournful relative who talks in inaudible whispers and looks like a funeral. Yet, if the one person whose companionship is at once cheering and soothing is favoured too much, offence is given to the others, and the unfortunate nurse is blamed. Again, the patient may be extremely fond of the friend whose visits are the most hurtful, and it requires all the nurse's tact and firmness to pursue the path she knows is right. A most troublesome mortal to manage is the sick man who is getting better, and who has decided he is well. The feverish symptoms have departed, and he is left with a moist and perspiring skin, comfortably tucked up in bed, while his nurse, who has hitherto had no reason to doubt his sanity, goes out for a little fresh air. On her return she is met on the door-mat by the entire household, who, with much pantomimic action, convey her to the damp office or chilly library, where the patient, in the airiest costume permissible, is discovered calmly checking accounts or searching for a favourite book.

The private nurse learns to listen with a sinking heart to such speeches as, "Maria has been with me twelve years," or, "John lived with my father, and is more like a friend than a servant," for she knows that these faithful old tyrants will probably look on her as an intruder, and do their best to make "that stuck-up young person know her place." In many cases the nurse is simply sent for to help these ancient domestics, who must on no account be put aside for a stranger. And she must do the fetching and carrying for them with a smiling face while they bungle over her work, till she can suggest that now they have put her in the right way they may as well take the opportunity of resting, or that household affairs may perhaps require their experienced and admirable supervision. Courtesy will always win with this class, and it is possible to enter into chaos and black looks and to emerge from among smiling faces, while the best the house can afford is hardly good enough for the whilom interloper. Nothing wins the hearts of servants so much as a little interest in the aches and pains to which they, like the rest of humanity, are liable. An inquiry after a headache, or the poulticing of a bad finger, will often be returned with piled-up interest. The hospital nurse is used to a certain routine; when there are a large number of patients to care for, work must be done at the appointed time. She is under the supervision of the Matron or the Ward Sister, and to these she can always turn when in a difficulty. Whatever she wants in the way of nursing apparatus is at hand, and of the best and most convenient kind. All this is changed at private cases, and it is easily understood why an experienced hospital nurse often succeeds so badly at private work; she has so much to unlearn, there is no one to take the responsibility, and the want of nursing utensils is bewildering. For years she has been in a groove, and it is very difficult to take comfortably to a life that requires adaptability and ingenuity. Two years at a good hospital are sufficient for a woman who intends to devote herself to private patients, and that most Matrons consider it long enough is made apparent by the fact, that they continually draft probationers of even less training into their private staffs. This is undoubtedly valuable to the nurse, as she is able to see both sides and can choose wisely which of two decidedly different careers best suits her. The good nurse is a true socialist, for it matters little to her whether her patient be a royal duke or a poverty-stricken clerk; she is as much at home in the palace of the millionaire as in the tenement of the poorest artisan, and little is she worthy of her calling if the latter gets one iota less care than the former. It is one of her curious experiences, that one week she is served from a silver salver by a liverid footman, and the next she may have to light the little fire that will cook at the same time her modest chop and the patient's broth. The houses of the rich are not always the pleasantest places for the nurse. When there are many servants with strictly defined duties, they are apt to dislike the extra work entailed by sickness; or you want some simple thing and are sent from valet to housemaid, from housekeeper to butler, till you long to flee away to a place where luxury does not supersede comfort. It is almost needless to say that nurses should never talk of the private affairs of their patients. Often it is a relief to the invalid to unburden the mind to a kind and sympathetic nurse. But it must be remembered that if it were not for sickness such confidences would not be given, and in many cases a morbid state of the nerves will give rise to exaggerations for which the patient is not responsible. As the cassock is stripped from the priest who betrays the secrets of the confessional, so should the nurse who betrays her patient be stripped of her uniform. The receiving of gifts from private patients has been for years a vexed question with the institutions, and probably when nurses were less educated than now a preventive rule was needed. An educated woman is rarely offered money, and those nurses who receive their own fees are not considered objects of charity. During seven years of private nursing I have once been offered a monetary gift, and that was during my one year at a private home, when an old patient placed a sum in the savings bank in my name, and asked me not to draw it unless necessity compelled me—it is there yet. That was before the days of the R.N.P.F. Little gifts of remembrance should be accepted, as a refusal often pains the kind donors, though it is sometimes difficult to receive gracefully a copy of "Early Piety" or a volume of sermons. Some of the private institution rules require altering, for instance that obnoxious reference to beer money that still disgraces the regulations of a few private homes. It is a degrading thought that there are many members of our profession who are supposed to require a small weekly sum as an incentive to temperance. The truth is that the institution nurse is so badly paid that she is glad to get the supplement to her salary even if it does contain a sting. It is a good rule that the nurse should not take her meals with the servants, but remembering the large section of capable women among us who are happier in the servants' hall than in their lonely rooms, I would like to add, unless she prefer it. Another thing is she gets a hotter and far more appetising meal by going downstairs. Often the servants are as well brought up as the nurse, and in many houses the linen is as fine and the table as well set as in the dining room. In one house I went to, where the servants were old and valued, but with strong prejudices, they all refused to carry my meals to my room, and it was finally settled that I should lunch and dine with the family and have breakfast and tea with the maid. I spent many happy months with those people, and now when I call there is a friendly rivalry as to whether I shall have tea in the drawing-room or the kitchen, and sometimes I have two lots. N.B.—The tea and cake downstairs are much the best.

### Amusements.

Miss Violet Westmacott's Dramatic and Musical Recital, held at the Steinway Hall on April 30th, in aid of the choir fund of the Kyrle Society, was, saving the absence of Mr. Lawrence Kellie, in every way a success. Miss Angela Vanborough's violin solos were remarkable for their excellence, spirit, and grace. Miss Mary Appach, who has an exceptionally high soprano voice of fine quality, especially in the upper register, sang three solos very effectively, and was especially happy in her choice of "Mia Piccirella" by Gomez. Miss Fisher showed proficiency in her pianoforte solos, and proved herself an excellent accompanist. Miss Violet Westmacott gave three recitations. In "The Martyr of the Arena," a piece suggested by Mr. Edwin Long's picture, "Diana or Christ?" she held the audience spellbound from start to finish. She has an excellent voice, is very quiet and effective in her style, and is altogether a performer of great promise and power. Mr. John Hare and Mr. Augustus Harris should look her up, as she would be invaluable in the case of more than one of the famous pieces the former has so successfully produced. The proceeds ought to add a substantial sum to the funds of the Kyrle Society, as there was a crowded and enthusiastic audience.

### Appointments.

Hull Royal Infirmary.—Miss Grace Hall has been appointed Night Superintendent of this institution; she trained at the Derbyshire General Infirmary, where she also had charge of wards, and has lately been engaged in private nursing for the West Kent Hospital at Maidstone.

Romford Cottage Hospital.—Miss E. A. Bostock has been appointed Nurse-Matron of this institution; she trained at the Marylebone Infirmary, and has acted as Charge Nurse at Newport Infirmary for the last eighteen months. She is succeeded at Newport by Mrs. Harriett Jones, late of Cardiff Infirmary.

Miss Abbott, who is leaving the Manchester Clinical Hospital, has been presented by the Committee with a writing table, by the medical staff with a reading lamp, by the nursing staff with a clock, by the present and past House Surgeons with a writing set; all with suitable inscriptions.

### To the Grumblers.

Don't begin to grumble,
Don't begin to fret;
Things may be are tiresome,
They'll be brighter yet.

Don't begin to fancy
Your's a grievous load,
There's a bigger burden
Just across the road.

Don't begin to worry
Over what "They say,"
Take your task and do it,
That's the better way.

Don't stand still to chatter
Of the last strange news,
Time is slipping past you,
Why the moments lose?

Don't begin to envy,
Jealousy is blind;
Keep the smiles before you,
Put the scowls behind.

Start the day by smiling,
Look for flowers and fun,
Though tired you'll still be cheerful
When the day is done.

### How to Rest.

WE end-of-the-century folk are in somewhat serious danger of losing a certain vital necessity to our well-being: the capacity for resting ourselves. Push onwards! Push upwards! Such war cries have been spurs applied too freely, and that it is so, we are beginning to see, as yet dimly, but still there is a glimmer of light, and presently a whole flood of knowledge will be let in upon us. Already our neighbours in America who beat us in bodily and mental swiftness, have lent their ears to a timely warning, that not only is everybody burning the candle at both ends, but some of us have set light to the middle of it in our frenzied hurry to get on in the world, and that an inevitable collapse of the structure of the human animal must ensue. For a year back a theory of salvation from such suicide has been simmering in the active brains across the "silver streak." Wise men, says the *Boston Herald* recently, have discovered that one of the hardest things that adult people have to do is to treat their minds and bodies according to the laws of nature; they have, therefore, set about studying the laws of repose for the nerves, and the laws of animal life for the control of physical forces. A remedy for the nervousness and the mental strain must be hit upon, and, of course, it is a woman's wit that has jumped to a conclusion, in the shape of a proposed course of training to set matters right. Miss Payson-Call has rendered a public service by giving to the world a book, "Power through Repose," which shows forth the working of the suggested system by which people are to learn to rest, with as complete relaxation of all their forces as is found in the repose of animals. The horse, the ox, the dog, and the cat, when not on duty, know how to rest. But human beings, we are told, are so often under the control of unnatural forces that they do not rest even when they sleep, to say nothing of the brief spells when they are not at work, and think they are at play. Particular attention is called to this fact, as a great change is going on at the present time in the understanding of what the mind and body require for the renewal of their powers. The severer methods of the gymnasium are, nowadays, beginning to be at a discount. The idea is not so much to develop muscle as it is to secure an equable training for the whole man.

The point Miss Payson-Call makes chiefly concerns the nerves; that is, the acquisition of power through the repose of the nervous system, which to quote her, "is, metaphorically speaking, to dispossess yourself of your head or arms or legs, so that they are no longer a part of you. . . . Take the will power out of them and let them rest. You transfer the strain from the nerves to the muscles. You make the unused parts of the body or mind do their proper part in the unity of mental and physical forces . . . . and the perfection of this plan" consists in reaching the "unity of life and its laws." This theory, a sufficiently admirable one, specially bears upon the habits of women, who, living under highly nervous conditions, lose their higher gifts, through inability to obtain genuine rest for mind and body. But to make the said theory practicable, it must be combined with the newer ideas of physical exercise, it being distinctly necessary that the nerves and the muscles and the mind should equally secure change and relaxation in their exercise. The mind needs the repose gained by physical exercise; the nerves need to be disconnected from the will; the muscles ought so to be exercised that the whole body is invigorated without being wearied. That is a result not always reached by the vigorous gymnastics so largely in use, but it is secured, we hear, by the principles set forth in the book before us, which bring every part of the body into a healthful and natural activity.

"If the mental strain of thousands of overtaxed men and women is to be taken off, it must be by knowing how to relax the tension of their nerves," argues the American writer. Therefore, the sooner the harassed and the weary make acquaintance with the new code of physical training, which consists of the use of such simple exercises as are within the reach and capacity of all, the better for the present fagged generation, and also for those generations of the future which, if something be not done, will in mind and body be but feeble, boneless shadows of our rapidly wearing-out selves.

### Presentations.

LAST week the nurses of the Queen Victoria Nursing Institution, at Wolverhampton, presented Miss Louisa Wenham with a beautiful marble timepiece, accompanied by a kind letter from Dr. Coleman, conveying the expression of their regret at her resigning the post of Lady Superintendent.

MANCHESTER SOUTHERN HOSPITAL.—Miss Abbott has been appointed Matron of this hospital. She trained at Bart.'s and Great Ormond Street, and for the last six years has been Sister at the Manchester Clinical Hospital. Her testimonials are excellent.

REFERENCE was made at the annual meeting of the Metropolitan and National Nursing Association for the Sick Poor, held at Grosvenor House, on May 1st, to the resignation by Miss E. M. Mansel of the Superintendentship of the Central Home, Bloomsbury Square, a post she has filled most admirably for the last ten years. Later in the afternoon at a less formal gathering at the home itself, at which a great number of the "old Bloomsbury nurses" trained by Miss Mansel were present, Mr. Warrington Haward (Dr. Cheadle being unavoidably detained), after a most appreciative speech, handed to her, in the name of the nurses, as a small token of their gratitude and love, a handsome gold watch, the subscription list for which was signed by about eighty names. The presentation being a complete surprise to Miss Mansel, she deputed Dr. Cheadle, who arrived at the moment, to return her thanks, and he added to this a most earnest expression of his own and his fellow-lecturers' high esteem for her, and their keen regret at her departure. Miss Mansel's valuable services are not to be lost to the cause of district nursing, as she has been chosen as one of the Inspectresses of the Queen Victoria's Jubilee Institute.

### Amusements and Relaxation.

**SPECIAL NOTICE TO CORRESPONDENTS.**

**Second Quarterly Word Competition commenced April 4th, ends June 27th, 1891.**

Competitors can enter for *all* quarterly competitions, but no competitor can take more than *one first prize* or *two* prizes of any kind during the year.

The words for dissection for this, the SIXTH week of the quarter, being

"ACCIDENT."

| Names. | April 30th. | Totals. | Names. | April 30th. | Totals. |
|---|---|---|---|---|---|
| Christie | 53 | 171 | M. G. | 50 | 148 |
| Patience | 52 | 167 | Ivanhoe | 51 | 147 |
| Agamemnon | 54 | 172 | Weta | 49 | 142 |
| Hope | 53 | 173 | Lady Betty | 50 | 164 |
| Reldas | 54 | 169 | Mortal | — | 76 |
| Lightowlers | 49 | 162 | Little Eliza | 47 | 142 |
| Nurse J. S. | 50 | 129 | Dore | — | 95 |
| Qu'appelle | 51 | [illegible] | Ladybird | 48 | 135 |
| Jenny Wren | 54 | 158 | Psyche | 52 | 143 |
| Wyameris | 51 | 168 | Ugug | 32 | 115 |
| Pa gnton | 47 | 142 | Harrie | — | 18 |
| Theta | 54 | 165 | Grannie | 38 | 124 |
| Success | — | 17 | Eale | 47 | 125 |
| Tired | 45 | 136 | Grimalkin | — | 53 |

For Rules see THE HOSPITAL, April 4th, 1891.

**Notice to Correspondents.**

N.B.—All letters referring to this page which do not arrive at 14[illegible] Strand, London, W.C., by the *first post on Thursdays*, and are not addressed PRIZE EDITOR, will in future be disqualified and disregarded.

## Figures and Tables

**Figure f1:**
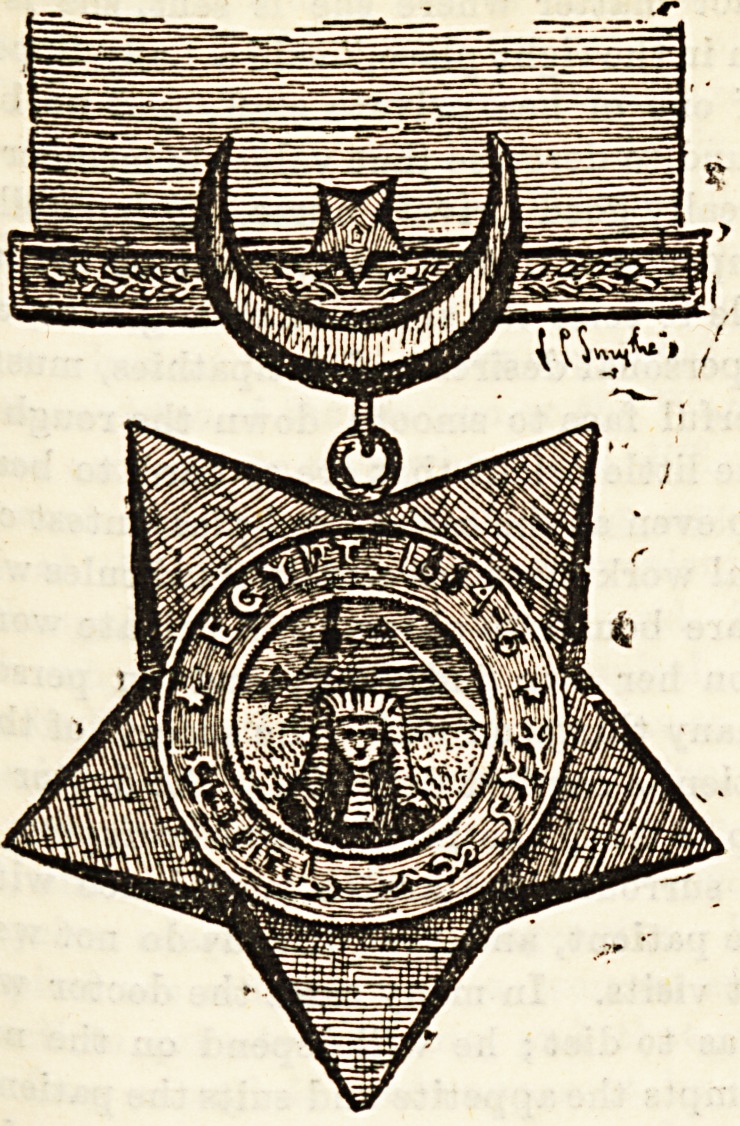


**Figure f2:**